# High resolution multislice imaging of the fetal heart using iGRASP and MOG

**DOI:** 10.1186/1532-429X-18-S1-P44

**Published:** 2016-01-27

**Authors:** Christopher Roy, Mike Seed, Christopher Macgowan

**Affiliations:** 1 grid.17063.33Medical Biophysics, University of Toronto, Toronto, ON Canada; 2 grid.42327.300000000404739646Physiology and Experimental Medicine, The Hospital for Sick Children, Toronto, ON Canada; 3 grid.42327.300000000404739646Division of Pediatric Cardiology and Diagnostic Imaging, The Hospital for Sick Children, Toronto, ON Canada

## Background

Multi-slice CINE MRI is an integral facet of postnatal cardiac examinations, but its application to the fetal heart is hampered by the lack of a fetal cardiac gating signal and the possibility of fetal motion. In this work, a method for reconstructing high spatial and temporal resolution multi-slice images of the fetal heart in a clinically acceptable scan time is presented. The proposed method combines metric optimized gating (MOG), a solution for fetal cardiac gating, with golden-angle radial sparse parallel MRI (iGRASP), a motion-robust acquisition and reconstruction scheme for highly undersampled data acquisitions [1, 2].

## Methods

Fetal acquisitions were performed using a 1.5T Avanto MRI system (Siemens, Germany). Ungated golden-angle radial spokes were acquired with scan parameters as follows: TR/TE = 5.7/2.7 ms, FA = 86°, field-of-view = 32 × 32 cm^2^ 640 spokes per slice, readout length 320, voxel = 1 × 1 × 4 mm^3^, scan length = 3.7 seconds/slice, reconstructed temporal resolution = 23 ms. For each slice, radial spokes were retrospectively sorted according to an estimation of the fetal cardiac cycle to create CINE frames of the beating fetal heart. The data were then iteratively resorted according the MOG method until a metric for image quality (entropy) was minimized. Each reconstructed CINE frame consisted of only 32 spokes, so a compressed sensing scheme (temporal total-variation) was used to suppress artifact [2]. Compared with the Nyquist sampling rate, the reconstructions corresponded to an acceleration rate of ~16. To cover the entire fetal heart, 20 slices were acquired with 50% overlap and the total scan time was 73 seconds.

## Results

**Figure 1:**
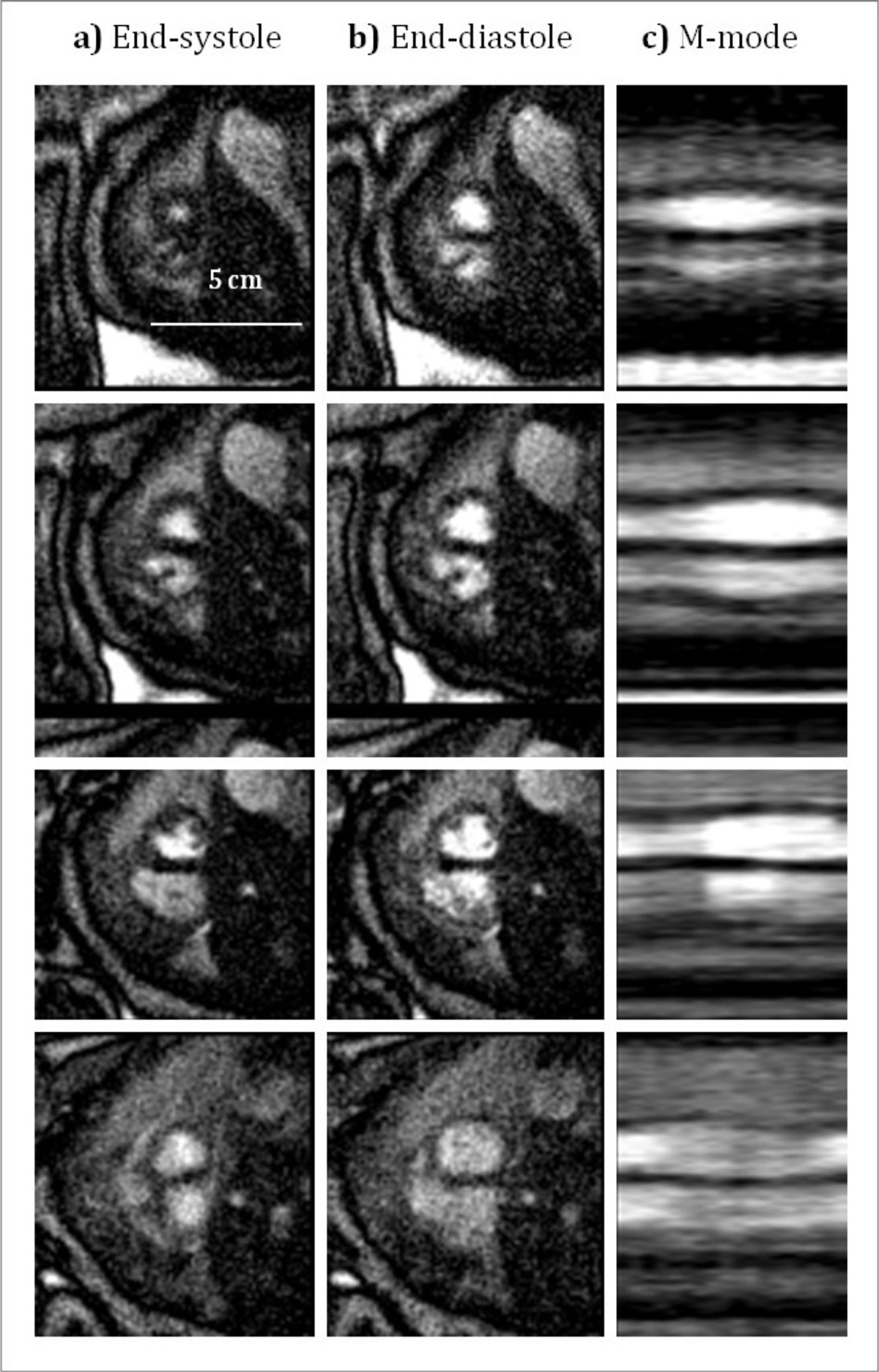
**Short axis images of the fetal heart. Four slice locations are shown during a) end-systole, b) end-diastole, and c) as an m-mode display through the middle of the left ventricle**.

**Figure 2:**
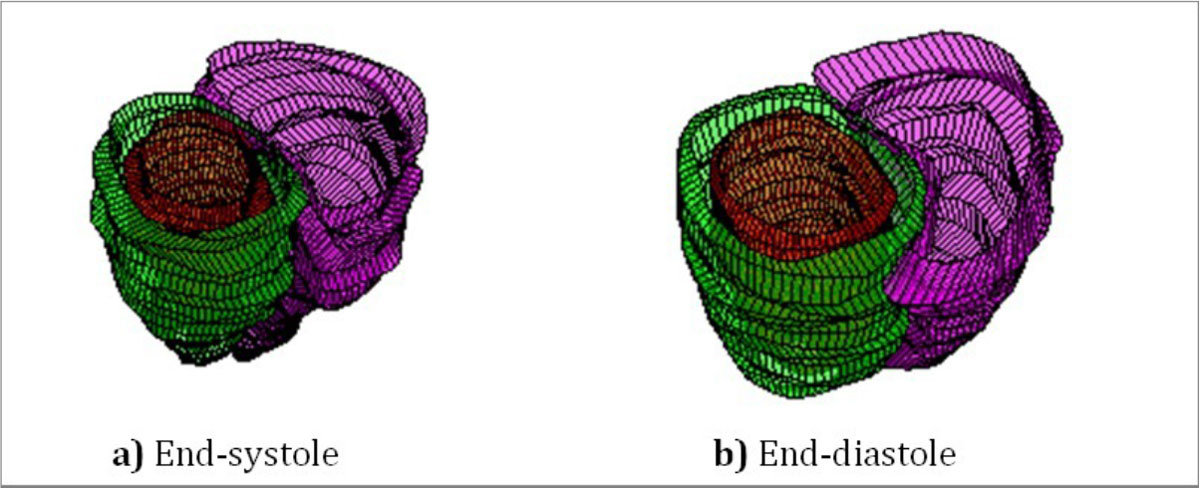
**Ventricular volume reconstructions of multi-slice images of the fetal heart during a) end-systole and b) end-diastole**.

Figure 1 shows four representative short-axis views of the fetal heart at end-systole and end-diastole, and an M-mode display of the temporal profile through the ventricles. Small cardiac structures such as the left ventricle papillary muscles are well demonstrated. Normal myocardial contraction is visible in the M-mode display. Assessment of cardiac function was possible through 3D ventricular volume reconstructions of the multi-slice stack producing a measured left ventricle ejection fraction of 51%. Figure 2 shows end-systolic and end-diastolic reconstructed volumes.

## Conclusions

Using iGRASP and MOG, high resolution multi-slice imaging of the human fetal heart was possible in a clinically acceptable scan time (73 seconds) despite the absence of conventional cardiac gating. We were able to identify moving structures of interest during radial contraction, thus capturing normal fetal cardiac motion. Volumetric reconstruction of multi-slice data allowed for quantitative assessment of cardiac function.
